# Gamma-Delta CAR-T Cells Show CAR-Directed and Independent Activity Against Leukemia

**DOI:** 10.3389/fimmu.2020.01347

**Published:** 2020-07-02

**Authors:** Meir Rozenbaum, Amilia Meir, Yarden Aharony, Orit Itzhaki, Jacob Schachter, Ilan Bank, Elad Jacoby, Michal J. Besser

**Affiliations:** ^1^Department of Clinical Microbiology and Immunology, Sackler School of Medicine, Tel Aviv University, Tel Aviv, Israel; ^2^Ella Lemelbaum Institute for Immuno Oncology, Sheba Medical Center, Ramat Gan, Israel; ^3^Center for Pediatric Cell Therapy, Sheba Medical Center, Tel Hashomer, Israel; ^4^Rheumatology Unit, Sheba Medical Center, Tel Hashomer, Israel; ^5^Division of Pediatric Hematology and Oncology, Sheba Medical Center, The Edmond and Lily Safra Children's Hospital, Tel Hashomer, Israel; ^6^Department of Pediatrics, Sackler School of Medicine, Tel Aviv University, Tel Aviv, Israel; ^7^Wohl Institute of Translational Medicine, Sheba Medical Center, Tel Aviv, Israel

**Keywords:** gamma-delta T cells, chimeric antigen receptor, leukemia, immuno oncology, B cell malignancies

## Abstract

Autologous T cells engineered to express a chimeric antigen receptor (CAR) against the CD19 antigen are in the frontline of contemporary hemato-oncology therapies, leading to high remission rates in B-cell malignancies. Although effective, major obstacles involve the complex and costly individualized manufacturing process, and CD19 target antigen loss or modulation leading to resistant and relapse following CAR therapy. A potential solution for these limitations is the use of donor-derived γδT cells as a CAR backbone. γδT cells lack allogenecity and are safely used in haploidentical transplants. Moreover, γδT cells are known to mediate natural anti-tumor responses. Here, we describe a 14-day production process initiated from peripheral-blood mononuclear cells, leading to a median 185-fold expansion of γδ T cells with high purity (>98% CD3+ and >99% γδTCR+). CAR transduction efficacy of γδ T cells was equally high when compared to standard CAR-T cells (60.5 ± 13.2 and 65.3 ± 18.3%, respectively). CD19-directed γδCAR-T cells were effective against CD19+ cell lines *in vitro* and in vivo, showing cytokine production, direct target killing, and clearance of bone marrow leukemic cells in an NSG model. Multiple injections of γδCAR-T cells and priming of mice with zoledronate lead to enhanced tumor reduction *in vivo*. Unlike standard CD19 CAR-T cells, γδCAR-T cells were able to target CD19 antigen negative leukemia cells, an effect that was enhanced after priming the cells with zoledronate. In conclusion, γδCAR-T cell production is feasible and leads to highly pure and efficient effector cells. γδCAR-T cell may provide a promising platform in the allogeneic setting, and may target leukemic cells also after antigen loss.

## Introduction

Transduction of T cells with a chimeric-antigen receptor (CAR) enables the targeting of extracellular domains, leading to CAR-induced T cell cytotoxicity, and cytokine production, in an MHC-independent manner (1). Autologous CAR-T cells directed against the pan-B-cell antigen CD19 have been approved for acute lymphoblastic leukemia (ALL) and non-Hodgkin lymphoma (NHL), after showing high remission rates in heavily pretreated patients, which in some may be durable (2, 3). Still, many patients do not respond to CAR-T cells or experience a relapse, through an orchestra of mechanisms including loss of transferred T cells in some patients and alterations in target antigen expression or density in others (4, 5). Thus, further multi-targeting approaches have been proposed, usually via dual CARs (5, 6), but sequential antigen loss has also been shown following CAR-mediated multi-targeting (7, 8). In addition these cellular therapies are still autologous products, necessitating complex, and individualized production associated with a significant financial burden.

Lymphocytes bearing the γδT cell receptor (γδT) are a small subset of peripheral blood cytotoxic T cells, which do not require antigenic presentation by MHC molecules for recognition and function (9, 10). *In vitro* and *in vivo* expansion of these cells is feasible, especially when exposing them to amino bisphosphonates such as zoledronate (11, 12). γδT cells are known to function across MHC-barriers, and do not cause graft-vs.-host disease (13). Moreover, anti-tumor activity has been demonstrated using expanded Vγ9Vδ2 T cells in preclinical studies and early phase clinical trials (14), though effects against ALL and NHL remain modest at most (13, 15, 16).

Since γδT cells can be safely applied in the allogeneic setting and exhibit natural anti-tumor reactivity, arming γδT cells with a CAR may provide a way to safely use allogeneic CARs and can potentially target minor clones with lower antigen density, which may not be eliminated by the standard CAR T cells. Here, we report our protocol to use γδ T lymphocytes as a platform for CAR-T cells. We show that γδCAR-T cells are effective against CD19 malignancies *in vitro* and *in vivo*, and have activity against leukemic clones lacking CD19 expression.

## Materials and Methods

### Ethics

Patient material was obtained as part of a clinical trial previously reported (NCT02772198) (17, 18) and approved by the Sheba Medical Center IRB and the Israeli Ministry of Health. All animal experiments were approved by Institutional Ethical Review Process Committees and were performed under Israel Institutional Animal care and use committee approval (1131/17/ANIM).

### Cell Culture

Leukemia cell lines Nalm6, CCRF-CEM, Toledo, and K562 were kindly provided by Steve Feldman, and grown in standard culture conditions, using RPMI medium supplemented with 10% fetal bovine serum (FBS), 2 mM L-Glutamate, Sodium Pyruvate, Hepes buffer 0.1 M (all from Biological Industries), and 100 U/mL penicillin and 100 μg/mL streptomycin (Sigma-Aldrich) (“target cell medium”). For activation and transduction, T cells were cultured in RPMI supplemented with 10% FBS, 2 mM L-Glu, 100 U/mL penicillin, and 100 μg/mL streptomycin and interleukin-2 (IL-2, 100 IU/ml, Novartis Proleukin) (“T cell medium”). 293T cells used for viral production were cultivated in DMEM high glucose medium supplemented with 10% FBS, 2 mM L-Glutamate, Sodium Pyruvate, Hepes buffer 0.1 M, and non-essential amino acids solution (all from Biological Industries). Alternatively, medium can be supplemented with human AB serum instead of FBS.

### CAR-T Cell Production

Peripheral-blood mononuclear cells (PBMCs) were isolated using centrifugation on LymphoprepTM density gradients (Alere technologies) and were activated in T cell medium with 100 IU/ml IL2 and either OKT3 50 ng/ml (Invitrogen) for standard T cell expansion (previously published) (17), or zoledronic acid 2.94 uM (Novartis) for γδT cell expansion. On day 5 of culture, activated cells were transduced with the CD19 CAR retrovirus, based on an MSGV backbone transduced with an FMC63-CD28-CD3zeta plasmid, kindly provided by Steve Feldman. For this purpose, non-tissue culture treated 6-well-plates (Falcon) were pre-coated with 20 ug Retronectin per well (10 ug/ml, Takara-Clontech) for 3 days at 4°C followed by a 20 min incubation with a 2.5% BSA solution (Caisson labs, BSA fraction V) in PBS (Biological Industries) and a single wash with PBS. Plates were loaded with 4 ml of virus diluted 1:1 with T cell medium per well and centrifuged at 2,000 g for 2 h at 32°C. Following centrifugation, the supernatant was collected leaving only 1 ml/well. Plates were then seeded with the OKT-3 or zoledronic acid activated PBMCs, between 2 and 2.5 × 106 cell/well, centrifuged at 1,000 g for 20 min at 32°C and incubated overnight at 37°C. Un-transduced γδT cells and standard T cells were treated the same way, just without addition of virus and served as negative control. On Day 9 γδT and γδCAR-T cells underwent αβTCR+ cell depletion using MACS LD depletion magnetic columns, FcR Blocking reagent, anti-TCRa/b-Biotin, and anti-biotin MicroBeads (all from Miltenyi). The remaining γδT cells and standard CAR-T cells were further expanded in IL-2 containing T cell medium until day 13–15.

Standard CAR T cells for the clinical setting were produced the same way as standard CAR T described here, with the only differences that clinical CAR T production utilizes 300 IU/ml IL-2 (instead of 100 IU/ml), transduction is performed on day 2 (instead of day 5) and human AB serum is supplemented to the culture medium (instead of FBS) (17).

### Co-culture, Cytokine Levels, and Cytotoxic Assays

Co-cultures were carried out in flat bottom 96-well-plates. For IFN-γ secretion tests T cells and targets were plated 100,000 cell/well each overnight in target cell medium. ELISA for the detection of human IFN-γ was carried out using Biolegend's Elisa MAX™ Deluxe kit. For cytotoxic assays, targets were stained by CellTrace™ Violet according to manufacturer's instructions and seeded 40,000 cells/well. T cells were then added at 160,000 cells/ml. After 2.5–3 h at 37°C, cells were collected from wells and stained for apoptosis by Annexin V-Cy5 reagent (Biovision). CellTrace positive cells were assessed for AnnexinV staining by flow cytometry.

### Flow Cytometry

Flow cytometry was performed on a Beckman Coulter's Gallios. For detection of the CD19 CAR receptor we used a biotinylated anti-mouse FAB as a primary antibody (Jackson), Mouse Gamma Globulin as a blocking reagent (Jackson) and Anti-Biotin-Viogreen as a secondary antibody (Miltenyi). The following antibodies were used for additional staining: Anti Human CD3 –FITC, Anti Human TCRa/b-APC, Anti Human TCRg/d-PE, Anti Human CD19-PE, Anti Human CD10-PE-Cy7, Anti Human CD45 viogreen, Anti mouse CD45 APC, and for dead cell exclusion Ghost Red 780 Viability Dye (all from Miltenyi). All antibodies were incubated with samples for 20 min at 4°C. Analysis was done using the FlowJo analysis software V10.

### Nalm6 CRISPR CD19 KO

We generated a Lenti virus expressing CAS9 and a sgRNA targeting CD19. The sgRNA sequence 5′-TGGAATGTTTCGGACCTAGGTGG-3′ (19) was cloned into pL-CRISPR.EFS.GFP (addgene Plasmid #57818 - Lentiviral CRISPR-Cas9 delivery for SpCas9 and sgRNA. Co-expresses eGFP via P2A cleavage site). Envelope plasmid was pMD2.G (addgene Plasmid #12259) and packaging plasmid psPAX2 (addgene Plasmid #12260). Plasmids were transfected to 293T cell line using the calcium Phosphate Profection Mammalian Transfection System (Promega cat#E1200). Viral supernatant was used for infection of Nalm6 cells with addition of Polybrene (8 ng/ml). CD19 positive cell were depleted using PE conjugated anti-CD19 antibody and anti-PE magnetic beads, on a MACS LD depletion column (Miltenyi), followed by single cell culture by plating. CD19 negative single-clones were confirmed by flow cytometry and wells were sequenced for the CD19 KO locus.

### Animals and *in vivo* Models

For all *in vivo* experiments, 8–15-weeks-old NOD-SCID-IL-2Rγ- (NSG) female mice, purchased from the Jackson laboratories, were used. Mice were tail-vein injected with 1 × 10^6^ Nalm6 cells for leukemia inoculation, followed by intravenous (IV) injections of effector cells with or without intraperitoneal (IP) injections of zoledronate (20).

### Statistical Analysis

All statistical analyses were performed using the Prism v8 (GraphPad Software). Statistical comparisons between two groups were determined by two-tailed parametric or non-parametric (Mann–Whitney *U*-test) *t*-tests for unpaired data or by two-tailed paired Student's *t*-tests for matched samples (produced from same donor). *P* < 0.05 were considered statistically significant.

## Results

### Generation of Human CD19 CAR Expressing γδT Cells From Peripheral Blood

**Figure 1:**
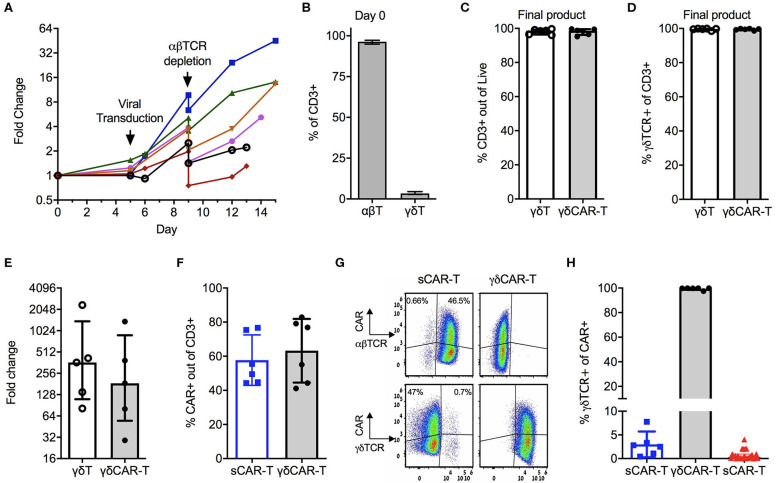
Production of γδCAR-T cells: **(A)** Production scheme, showing total cell expansion (by fold change) along the protocol. Day 0 is the collection of blood, PBMCs isolation, and T cell activation. Each line represents a different healthy donor. **(B)** γδ-T/ αβ-T cells composition of donors' blood out of CD3 positive cells (*n* = 5). **(C)** CD3 positive cells in the final product of un-transduced γδ-T and transduced γδCAR-T cells (*n* = 6). **(D)** Purity of γδTCR+ cells in the final product of both protocols (*n* = 6). **(E)** γδ-T cells fold change expansion during the γδCAR-T production protocol (*n* = 5). **(F)** CAR transduction efficiency by flow cytometry gated on CD3 positive cells, of the standard CAR (sCAR) and γδCAR-T cell products (*n* = 6). **(G)** Dot plots of a representative sample showing CAR expression in γδ and αβ-T cells populations in the final product of sCAR and γδCAR-T cell protocols. **(H)** γδTCR positive cells gated on CAR positive cells in the final composition of sCAR-T cells (blue squares, *n* = 6), γδCAR-T cells production protocol (black circles, *n* = 6) and the clinically manufactured sCAR-T cells (red triangles, *n* = 25). Bars are at the median value, and error bars represent interquartile range.

We first devised and calibrated a protocol for the generation of γδT cells expressing the CD19 CAR and depletion of αβ-TCR+ cells (Figure 1A). On average, γδT cells consisted of 3.4% (± 0.73%) of CD3-positive cells in the initial starting material of PBMCs (Figure 1B). Activation with Zoledronate on day 0 led to specific proliferation of γδT cells, whilst the total number of cell remained similar. On Day 5, cells were transduced with the CD19 CAR, followed by αβTCR+ depletion on day 9 and further proliferation of γδCAR-T for a total of 13–15 days. Un-transduced γδT cells served as control. The final products of transduced as well as un-transduced γδT cells contained 98% (±1.77%) and 98.1% (±1.49%) CD3 positive cells, respectively, with high purity of γδT cells, accounting for 99.5% (±0.5%) of the CD3+ cells (*n* = 6, Figures 1C,D). The median fold change of the γδT cells was 185 (range, 29–1,376) for transduced cells compared with a median of 363 (range, 81–2,350) for un-transduced γδT cells, with a variable range between different donors (Figure 1E, *p* = 0.2 paired *T*-test). As controls, we ran in parallel a standard CAR-T cell (sCAR-T) production for each donor, using a protocol as for the clinical setting (17), just in a smaller scale and transduction performed on day 5 instead of 2. CAR transduction efficacy ranged between 40 and 80% (Figure 1F), and did not differ between sCAR-T and γδCAR-T (60.5% ± 13.2 and 65.3% ± 18.3, respectively). A representative FACS dot plot is presented in Figure 1G. Of note, the sCAR-T cell product had also γδT cells and γδCAR-T cells (Figures 1G,H). We analyzed 25 infusion products administered to leukemia and lymphoma patients enrolled on a clinical trial for γδTCR expression. Final products had an average of 1.15% γδT and 0.77% γδCAR-T (Figure 1G). The, percentage of γδCAR-T in the final product was not associated with response (17). To summarize, using this protocol we are able to produce a pure fraction of γδT cells lacking αβ T cells, with high expression of the CD19 CAR, and this within a course of 2 weeks.

### γδCAR-T Cells Show CD19 Dependent Activity Against Tumor Cell Lines

**Figure 2:**
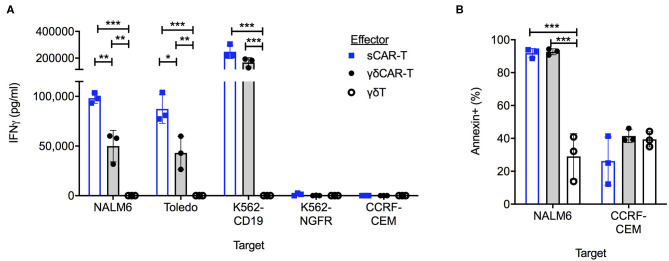
γδCAR-T cells demonstrate CD19 target dependent activity. **(A)** IFNγ levels in co-culture medium of listed effectors with CD19 positive and negative target cells. **(B)** Cytotoxic activity of effector cells against CD19 positive Nalm6 cells and CD19 negative CCRF-CEM cells. Cell trace violette pre-stained targets were evaluated for Annexin binding by FACS analysis. Blue bars, sCAR; Gray bars, γδCAR-T; White bars, γδ-T cells. *P ≤ 0.05, **P ≤ 0.01, ***P ≤ 0.001, Two tailed paired *T*-test. Error bars represent standard deviation.

To test for the efficacy of the γδCAR-T cells in comparison to the sCAR-T cells *in vitro*, co-culture assays against CD19 positive and negative cell lines were performed. Un-transduced activated γδT cells, γδCAR-T cells, and sCAR-T cell were co-incubated with the B-ALL cell line Nalm6, the B-NHL cell line Toledo, and with K562 transduced to express CD19 (K562-CD19). Antigen-negative controls were the T-ALL cell line CCRF-CEM and K562-NGFR cell line. Testing for IFNγ secretion after over-night incubation at a T cell/target ratio of 1:1 revealed γδCAR-T cells are highly reactive against CD19 expressing tumor cells (Figure 2A). γδCAR-T and sCAR-T cells exhibited substantially higher levels of CD19 dependent IFNγ secretion relative to un-transduced γδT (γδCAR-T vs. γδT, *p* = 0.005, *p* = 0.01, and *p* = 0.001; sCAR-T vs. γδT, *p* < 0.001, *p* < 0.001, and *p* = 0.001, for NALM6, Toledo, and K562-CD19, respectively). The level of IFNγ in the supernatant of co-cultures with γδCAR-T cells was lower than measured in the co-culture with sCAR-T cells in the case of Nalm6 and Toledo cell lines (*p* = 0.007 and *p* = 0.02, respectively), but not with the artificially-expressing K562-CD19 cell line (*p* = 0.08, Figure 2A).

In order to test for cytotoxic activity, targets were evaluated for annexin staining after a co-culture for 2.5 h at a T cell/target ratio of 5:1. Both γδCAR-T and sCAR-T cells demonstrate enhanced cytotoxic activity against the CD19 positive Nalm6, leading to 92% (±2.8%), and 93% (±1.8%) annexin expression on targets in comparison to activated γδT cells (29 ± 13.8%, *n* = 3, *p* < 0.001). No difference was seen in annexin expression of CD19 negative targets (Figure 2B).

### γδCAR-T Cells Show Enhanced *in vitro* Cytotoxicity Against CD19 Negative Target Cells

**Figure 3:**
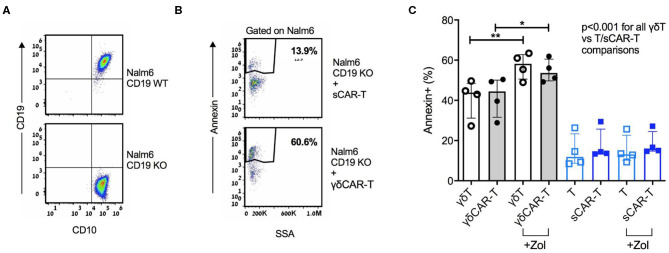
γδCAR-T cells demonstrate activity against CD19 negative clones. **(A)** Generation of Nalm6 CD19 KO cells via CRISPR yields CD10 positive CD19 negative cells (Nalm6^19neg^). **(B,C)** Activity of γδCAR-T cells against Nalm6^19neg^ cells is further enhanced by priming the target cells with zoledronate. **(B)** Dot plot representation showing Annexin staining of targets cells (cell-trace violet stained Nalm6^19neg^) co-cultured with sCAR-T cells (top) or γδCAR-T cells (bottom). **(C)** Specific cytotoxicity of different effectors against Nalm6^19neg^ cells. γδ-T and γδCAR-T cells in gray bars, sT, and sCAR-T cells in blue bars. Un-transduced cells in unstained circles and CAR-T cells in filled circles. *P ≤ 0.05, **P ≤ 0.01, Two tailed paired *T*-test. Error bars represent interquartile range.

A major barrier of CAR-T therapy is loss of target antigen, by various mechanisms. γδT cells are known to exert an anti-leukemic activity, especially after priming with zoledronate (13). Thus, we hypothesized that utilizing non-specific mechanisms may result in cytotoxicity also against CD19-negative targets. To further investigate this finding, we generated a CD19 knock-out B-ALL cell line Nalm6 (Figure 3A). γδCAR-T, γδT, sCAR-T, and un-transduced activated T cells were co-cultured with Nalm6^19neg^ for a period of 3 h at a T cell:target ratio of 4:1. Both γδT cells and γδCAR-T cells demonstrated enhanced cytotoxicity against Nalm6^19neg^ cells compared to standard T cells or sCAR-T cells (Figures 3B,C, *p* < 0.001 for all comparisons of γδT or γδCAR-T vs. un-transduced T or sCAR-T). Priming with Zoledronate further enhanced this effect, leading to higher target annexin expression of Nalm6^19neg^ cells incubated with γδT (57% after priming vs. 41% without zoledronate, *p* = 0.007) as well as γδCAR-T (55% after priming vs. 42% without zoledronate, *p* = 0.02, Figure 3C), eluding to a potential effect that may target minor CD19-negative clones.

### γδCAR-T Cells Exhibit *in vivo* Activity Against Tumor Cell Lines

**Figure 4:**
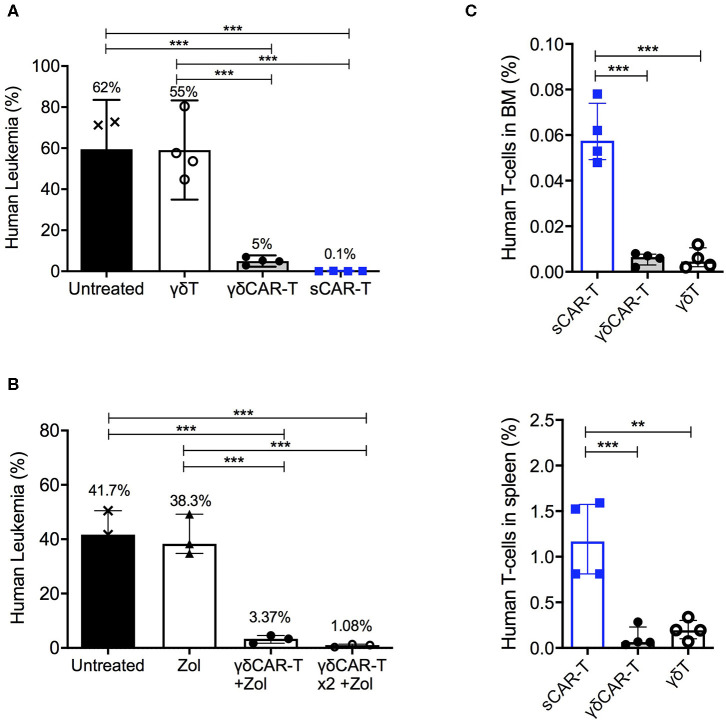
*In vivo* activity of γδCAR-T cells. **(A)** Human leukemia (Nalm6) gated on CD45+ CD10+ in the bone marrow of NSG-mice either untreated or treated with un-transduced γδT cells, γδCAR-T, or sCAR-T cells after 14 days. **(B)** Human leukemia (Nalm6) gated on CD45+ CD10+ in the bone marrow of NSG-mice either untreated or treated with zoledronate (Zol) alone, Zol with γδCART cells, or repeated dose of Zol and γδCAR-T. **(C)** Human effector cell persistence of.i.v. injected sCAR-T, γδCAR-T, and un-transduced γδ-T cells in mice bone marrow (upper panel) and spleen (lower panel), 3 days post injection. **P ≤ 0.01, ***P ≤ 0.001, Two tailed paired *T*-test. Bars are at the median value, and error bars represent interquartile range.

To investigate the *in vivo* efficacy of γδCAR-T, NSG mice were tail-vein injected with 1 × 10^6^ Nalm6 cells, and treated on day 2 with either 4 × 10^6^ γδCAR-T cells, sCAR-T, or γδT cells per mouse. CAR transduction efficacy was 75–85% in all experiments. Non-treated leukemia-bearing mice served as controls. Two weeks after the injection of effector cells, mice were sacrificed for evaluation of leukemic involvement of the bone marrow. The leukemic burden in the bone marrow of non-treated mice and of γδT cell treated mice was substantial (a median of 62 and 55% of bone marrow cells, respectively). The presence of human un-transduced γδT cells did not reduce significantly leukemia in this model (*p* = 0.89, Mann-Whitney). Treatment with either γδCAR-T or sCAR-T cells lead both to a drastic reduction in the leukemic burden in the bone marrow of recipient mice (to 5 and 0.1%, respectively, *p* < 0.001 compared to untreated or γδT cell treated mice), demonstrating *in vivo* activity of γδCAR-T cells (Figure 4A). Nevertheless, traces of leukemia were noted to be higher in recipients of γδCAR-T cells in comparison with the sCAR-T treated mice. In an intent to further improve the *in vivo* anti-tumor reactivity of γδCAR T cells, mice were conditioned with zoledronate, known to prime targets of γδT cells, and a second dose of γδCAR-T cells was administrated, both previously shown to improve anti-tumor effect of un-transduced γδT cells (21). Intraperitoneal injection of zoledronate (1.0 ug/gr) to leukemia-bearing mice on days −1, 2, 6, 8, and 10, still resulted in a median of 3% remaining leukemic cells in the bone marrow (Figure 4B). However, adding a second dose of γδCAR-T cells on day 7, 3 × 10^6^ T cells per mouse, in addition to zoledronate administration regimen lead to further reduction in Nalm6 burden in the marrow to almost 1% (*p* = 0.06, Figure 4B).

Since early loss of effector T cells may be associated with target-positive relapse, we next tested the persistence of the infused effector cells within the mice. Cell-trace violet stained γδT, γδCAR-T, and sCAR-T cells (5 × 10^6^ per mouse) were injected to leukemic mice. After 3 days, bone marrow and spleen were harvested and tested for presence of dye positive cells (Figure 4C). Lower number of human effector T cells were found in both the spleen and the bone marrow of murine recipients of γδCAR-T and γδT cells compared to recipients of sCAR-T cells (*p* < 0.001 for both comparisons in the bone marrow and *p* < 0.01 for both comparisons in the spleen). Thus, in the NSG model, we noted good anti-leukemic activity but limited persistence of γδCAR-T cells.

## Discussion

In this work we demonstrated the ability to effectively transduce and expand γδT cells with a CAR targeting CD19. γδCAR-T were effective against CD19+ tumor cell lines, both *in vivo* and *in vitro*. Moreover, we could see an effect against CD19- clones, which was CAR-independent.

Lack of allogenicity and potential of 3rd party-use make γδ cells excellent candidates as a CAR-T cell backbone. Attempts of γδT cell transduction with CARs have been previously reported, via zoledronate-based expansion of 1st-generation CARs (15) or proliferation of polyclonal γδT cells transduced with CD19 on an antigen-presenting cells (22). Both methods include a selection process, to ensure purity of the γδT cells, similar to our protocol. Of note, both methods showed *in vitro* efficacy against CAR target, but this was not compared to standard CAR-T cells, which in current days of commercially available CAR-T cells is essential. We preformed head-to-head comparison of the γδCAR-T to sCAR-T, showing comparable transduction efficacy and *in vitro* cytotoxicity against CD19 positive targets, and an *in vivo* effect that was profound, but inferior to that of sCAR-T. We also demonstrated the presence of γδCAR-T cells in clinical products, though at low percentages. The Sadelain group has previously showed comparable activity of γδCAR-T and sCAR-T *in vitro* and *in vivo*, against an intraperitoneal Raji tumor model (23). Raji, a Burkitt-NHL cell line, is known to express co-stimulatory molecules (24), which may have assisted *in vivo* killing by the γδCAR-T cells. Similar to others (22), we too could not show complete clearance of the aggressive Nalm6 ALL cell line in murine models treated with γδCAR-T cells, which might be explained by the limited persistence of γδCAR-T cells in comparison to sCAR-T cells. Indeed repeated infusion of the γδCAR-T improved anti-leukemic results. Loss of γδCAR-T may be a result of several factors, including lack of a supporting microenvironment for these cells in an immune-suppressed mouse, which may be improved with cytokine supplementation such as IL-2 (25). Another possible contribution may be due to increased activation-induced cell death (AICD) known to occur with activated γδT cells. These challenges should be addressed in further work.

The loss of CD19 is a major problem in relapsed patients with persisting CAR-T cells, or patients with prior CD19-directed therapy (26). Current models of using two (or more) CARs transduced on a single cell (by various methods) (27) show some success in preclinical models, but clinical results have not yet matured. Also, sequential antigen loss has been shown in patients with NHL or ALL. Utilizing the natural non-MHC restricted targeting optional by γδT cells has shown anti-leukemic activity, which can be enhanced after priming with zoledronate (13). We showed that this effect is retained after CAR transduction of γδT cells, and is independent of CAR activation by its ligand, as demonstrated by targeting antigen-negative cells. Thus, exploiting this non-specific MHC-independent targeting mechanism on top of the CAR specificity may prevent antigen loss and subsequent relapse and requires further investigation.

In conclusion, we established a rapid and robust protocol for γδCAR-T cell production. These cells demonstrated anti-tumor activity *in vitro* and *in vivo*. γδCAR-T cell may provide a promising platform in the allogeneic setting, and may target antigen-negative clones. Further challenges, including improving *in vivo* persistence, are to be addressed prior to clinical application. The equations should be inserted in editable format from the equation editor.

## Data Availability Statement

The raw data supporting the conclusions of this article will be made available by the authors, without undue reservation.

## Ethics Statement

The animal study was reviewed and approved by institutional ethical review process committee.

## Author Contributions

MR, EJ, IB, and MB contributed conception and design of the study. MR, AM, YA, and OI performed experiments and acquired the data. MR wrote the first draft of the manuscript. EJ, IB, JS, and MB revised it critically for important intellectual content. All authors contributed to manuscript revision, read, and approved the submitted version.

## Conflict of Interest

The authors declare that the research was conducted in the absence of any commercial or financial relationships that could be construed as a potential conflict of interest.
